# The Role of Augmented Reality in Surgical Training: A Systematic Review

**DOI:** 10.1177/15533506221140506

**Published:** 2022-11-22

**Authors:** Dhivya Suresh, Abdullatif Aydin, Stuart James, Kamran Ahmed, Prokar Dasgupta

**Affiliations:** 1 Guy’s, King’s and St Thomas’ School of Medical Education, 4616 King’s College London, London, UK; 2 MRC Centre for Transplantation, Guy’s Hospital, 4616 King’s College London, London, UK; 3 Department of General Surgery, 156446 Princess Royal University Hospital, London, UK

**Keywords:** surgical education, simulation, neurosurgery, orthopedic surgery, urology, vascular surgery, gynecologic laparoscopy

## Abstract

**Aims:**

This review aims to provide an update on the role of augmented reality (AR) in surgical training and investigate whether the use of AR improves performance measures compared to traditional approaches in surgical trainees.

**Methods:**

PUBMED, EMBASE, Google Scholar, Cochrane Library, British Library and Science Direct were searched following PRIMSA guidelines. All English language original studies pertaining to AR in surgical training were eligible for inclusion. Qualitative analysis was performed and results were categorised according to simulator models, subsequently being evaluated using Messick’s framework for validity and McGaghie’s translational outcomes for simulation-based learning.

**Results:**

Of the 1132 results retrieved, 45 were included in the study. 29 platforms were identified, with the highest ‘level of effectiveness’ recorded as 3. In terms of validity parameters, 10 AR models received a strong ‘content validity’ score of 2.15 models had a ‘response processes’ score ≥ 1. ‘Internal structure’ and ‘consequences’ were largely not discussed. ‘Relations to other variables’ was the best assessed criterion, with 9 platforms achieving a high score of 2. Overall, the Microsoft HoloLens received the highest level of recommendation for both validity and level of effectiveness.

**Conclusions:**

Augmented reality in surgical education is feasible and effective as an adjunct to traditional training. The Microsoft HoloLens has shown the most promising results across all parameters and produced improved performance measures in surgical trainees. In terms of the other simulator models, further research is required with stronger study designs, in order to validate the use of AR in surgical training.

## Introduction

The advent of the digital revolution has ushered forward an era where the traditional “see one, do one, teach one” method of surgical training is near obsoletion. Technological advances in the past decade have allowed digital simulation to arrive at the forefront of training and education, allowing for a limitless cycle of repetition and feedback for skill acquisition.

Simulation is defined as “encompassing any activity which aims to imitate a system or environment with the aim of assessing, informing and modifying behaviour” by ASiT (Association of Surgeons in Training).^1^ Surgical training, as much as any form of training, stands to benefit from virtual reality simulation, where a safe environment is established to practise skills without harm to others and to limit the drain on resources. However, surgery is a field where tactile feedback is imperative, which virtual reality alone cannot offer.^2^

Augmented reality (AR) is an alternative to Virtual Reality (VR) that allows for haptic feedback and interaction within a virtual environment. Where VR provides a completely immersive digital environment, AR overlays digital information over the physical world, hence combining the benefits of traditional ‘gold standard’ tactile training methods and VR.

Another alternative to VR is the emerging mixed reality (MR) technology. MR is similar to AR in that it overlays digital information over the physical world. While the 2 are often referred to interchangeably, the difference lies in the fact that MR is an extension of AR which allows for real-time interaction between the digital and physical elements. For the purposes of this study, it was decided to focus on AR. While MR has promising prospects, it is still a relatively new technology and is more loosely defined than AR and VR. The degree to which a digital environment can be extended in MR is much more advanced and overlaps in the literature with both AR and VR.^60,61^ This study has chosen to focus on AR rather than MR, as the inclusion of MR may introduce too much heterogeneity between the capabilities of each platform.

This study aims to evaluate the role of AR in surgical training and provide an update on the validity and effectiveness of the current AR training systems in use. Specifically, it aims to investigate whether the involvement of AR in surgical training produces better performance measures compared to other, more traditional training approaches.

The terms ‘better performance measures’ and ‘more traditional methods’ were left deliberately broad to account for the heterogeneity of the measures described in the literature.

## Materials and Methods

Prior to conduction of this review, the international prospective register of systematic reviews, ‘PROSPERO’, was searched to avoid duplication. The systematic review was carried out following the PRISMA (Preferred Reporting Items for Systematic Reviews and Meta-Analyses) guidelines.^3^

### Search Methods

A systematic search was performed in PubMed, Ovid (EMBASE) and Google Scholar using the search string: (“augmented reality” OR “augmented virtual reality”) AND (educat* OR simulat* OR train*) AND (“surgery” OR “surgical”). Cochrane (CENTRAL), Science Direct and the British Library databases were also searched to cover grey literature. The latest search was returned and finalised on February 2021. A manual search of the references used in the screened results and relevant reviews was carried out to identify any missing eligible studies.

### Study Eligibility Criteria

All original, English language studies describing augmented reality in the context of surgical education were eligible for inclusion. Studies with participants at any level of surgical training were accepted (including medical students), across all specialties in both clinical and non-clinical settings, so long as a clearly defined control and educational performance measure were identified. Studies were excluded if they: (i) were not conducted in a human surgical discipline; (ii) were describing the AR in the context of patient education; (iii) were only describing technological aspects and development of the AR model; (iv) had an unclear distinction between AR and VR; (v) if they did not include a control (vi) if they did not include the use of AR in a training/educational context or (vii) if they did not provide a clearly defined educational or performance measure.

### Data Extraction

All results were first screened by title and abstract. The remaining results and any studies deemed ‘dubious’ were examined in full. Data extracted from the eligible studies included the study design, the number of participants, the details of the AR simulator, and the training tasks and specialty area. Performance and educational outcomes were noted to determine the level of effectiveness and validity of the AR simulation for surgical training.

### Data Analysis

Upon selection, the studies were grouped according to the area of surgical training they addressed and subsequently assessed for validity and level of effectiveness. Due to the heterogeneity of the study designs, qualitative analysis was performed. Messick’s ‘the modern concept of validity’ conceptualised a framework that defines validity according to 5 parameters; content, response processes, internal structure, relations to other variables, and consequences.^4^ This was later expanded upon by Beckman et al.,^5^ who proposed a comprehensive rating scale to measure the strength of each validity parameter (Table A1).

McGaghie et al.^6^ outlined simulation-based translational outcomes to determine the ‘level of effectiveness’ (LoE), or ‘translational level’ (T) of a training model. These translational levels were used to describe gradated educational effects of a simulator model, beginning at the classroom or simulator lab level (T1), leading onto downstream effects of safer patient care approaches (T2), resulting in improved patient outcomes (T3), before moving onto the collateral, systemic effects, such as cost saving and skill retention (T4). For the purposes of this study, an adapted version of McGaghie’s translational outcomes was used, wherein T1 - which refers to knowledge, skills and attitudes - was divided into LoE 1 and LoE 2. LoE 1 referred to participants’ satisfaction with the simulator tool, while LoE 2 referred to development of knowledge and skills as measured by the simulator tool. Thus, the maximum LoE a study could be awarded was 5 (see Table B1 for more detailed explanation of LoE scores).

The validity ratings and translational outcomes for each study was initially scored by DS and then reviewed individually by AA. If both reviewers could not unanimously agree on the score for each parameter, a third reviewer (SJ) was consulted for majority consensus. For the purposes of the data analysis, the overall scores for each modality was determined by the highest score in each parameter across all the studies performed (Table 1).

**Table 1.** Analysis of results-validity and level of effectiveness.

Model	Reference	Specialty/area of training	Study design	Control used	Sample size/participants	Validity rating	LOE
Microsoft Hololens (Microsoft Corp, Redmond, WA, USA)	Al Janabi et al.^7^	Urology	Prospective observational	Conventional monitor	n = 72 (medical students, urology trainees and specialists)	Content: N	2
						Response processes: 1	
						Internal structure: N	
						Relations to other variables: 1	
						Consequences: N	
	Baum et al.^8^	Neurosurgery	Prospective observational	‘2D method’ conventional monitor	n = 51 (medical students, neurosurgery residents and attendings)	Content: 2	3
						Response processes: 0	
						Internal structure: 1	
						Relations to other variables: 0	
						Consequences: 2	
	Heinrich et al.^9^	Basic skills	Prospective observational	Box trainer	n = 8 (residents and consultants)	Content: N	2
						Response processes: 1	
						Internal structure: N	
						Relations to other variables: 1	
						Consequences: 0	
	Logishetty et al.^10^	Orthopaedics	Randomised control trial	One-to-one training from surgeon	n = 24 (medical students)	Content: 1	2
						Response processes: 1	
						Internal structure: N	
						Relations to other variables: N	
						Consequences: 1	
	Siff and Mehta^11^	Gynaecology	Prospective observational	Traditional self-study	n = 18 (gynaecology residents and fellows)	Content: 1	1
						Response processes: 1	
						Internal structure: N	
						Relations to other variables: N	
						Consequences: 0	
	Triberti et al.^12^	Cardiothoracic	Prospective observational	CT scan	n = 11 (medical students)	Content: 1	1
						Response processes: N	
						Internal structure: N	
						Relations to other variables: 0	
						Consequences: 1	
	Wang et al.^13^	Trauma	Prospective observational	Conventional telemedicine setup	n = 24 (medical students)	Content: 1	2
						Response processes: 0	
						Internal structure: N	
						Relations to other variables: 0	
						Consequences: 1	
System for telementoring with augmented reality (STAR) and	Rojas-Muñoz et al.^14^	Trauma	Prospective observational	Traditional self-study	n = 20 (medical students and surgical residents)	Content: 1	2
						Response processes: N	
						Internal structure: N	
						Relations to other variables: 2	
						Consequences: 1	
	Rojas-Munoz et al.^15^	Basic skills	Randomised control trial	Audio-only telemonitoring set up	n = 19 (first responders)	Content: N	2
						Response processes: 2	
						Internal structure: 1	
						Relations to other variables: 2	
						Consequences: 1	
System for telementoring with augmented reality (STAR)	Andersen et al.^16^	Trauma	Prospective observational study	Conventional telestrator	n = 20 (premedical trainees)	Content: 1	2
						Response processes: N	
						Internal structure: N	
						Relations to other variables: N	
						Consequences: 0	
	Andersen et al.^17^	Basic skills	Prospective observational study	Conventional telementoring	n = 20 (premedical students and medical students)	Content: 1	2
						Response processes: 0	
						Internal structure: 1	
						Relations to other variables: N	
						Consequences: N	
ProMIS AR simulator (Haptica, Dublin, Ireland)	Botden et al.^18^	Basic skills	Prospective observational	Box trainer	n = 90 (medical students)	Content: N	1
						Response processes: 1	
						Internal structure: 1	
						Relations to other variables: 1	
						Consequences: N	
	Feifer et al.^19^	Basic skills	Prospective observational	Box trainer	n = 15 (medical students)	Content: N	2
						Response processes: 0	
						Internal structure: 0	
						Relations to other variables: 0	
						Consequences: N	
	Leblanc et al.^20^	Basic skills	Prospective observational	Cadaver	n = 35 (postgraduate surgical trainees)	Content: N	2
						Response processes: 0	
						Internal structure: N	
						Relations to other variables: 1	
						Consequences: 1	
	Plymale et al.^21^	Basic skills	Prospective observational	Traditional self-study	n = 26 (medical students, residents and paediatric surgeons)	Content: 2	3
						Response processes: N	
						Internal structure: 0	
						Relations to other variables: 1	
						Consequences: 0	
ImmersiveTouch system (ImmersiveTouch, Inc, Universityof Illinois, Chicago, IL, USA)	Yudkowsky et al.^22^	Neurology	Prospective observational	Traditional self-study	n = 16 (neurosurgical residents)	Content: N	3
						Response processes: N	
						Internal structure: 0	
						Relations to other variables: 2	
						Consequences: 1	
	Luciano et al.^23^	Neurosurgery	Prospective observational	Traditional self-study	n = 51 (neurosurgical fellows and residents)	Content: 1	2
						Response processes: N	
						Internal structure: 0	
						Relations to other variables: 0	
						Consequences: 1	
	Luciano^24^	Neurosurgery	Prospective observational	Procedure performed in clinical setting	n = 196 (surgical fellows and residents)	Content: 2	2
						Response processes: N	
						Internal structure: 2	
						Relations to other variables: 1	
						Consequences: 1	
Google Glass (Google Inc, mountain View, CA, USA)	Borgmann et al.^25^	Urology	Prospective observational	Standard procedure performed in clinical setting	n = 31 (broad certified urology residents)	Content: N	3
						Response processes: 0	
						Internal structure: N	
						Relations to other variables: N	
						Consequences: 0	
	Dickey et al.^26^	Urology	Prospective observational	Standard procedure performed in clinical setting	n = 30 (urology residents and fellows)	Content: N	1
						Response processes: N	
						Internal structure: N	
						Relations to other variables: 1	
						Consequences: 0	
	Peden et al.^27^	Basic skills	Randomised control trial	Wet lab	n = 14 (medical students)	Content: N	2
						Response processes: 1	
						Internal structure: 1	
						Relations to other variables: N	
						Consequences: 0	
Perk Station (National Alliance for Medical Image Computing (NAMIC), Boston, MA, USA)	Yeo et al.^28^	Orthopaedics	Randomised control trail	Classical freehand insertions	n = 40 (medical students and residents)	Content: 1	2
						Response processes: 1	
						Internal structure: N	
						Relations to other variables: N	
						Consequences: 1	
Perk Tutor (Queen’s University, Kingston, ON, Canada)	Keri et al.^29^	Neurosurgery	Randomised control trial	Ultrasound display	n = 24 (residents)	Content: 1	2
						Response processes: 1	
						Internal structure: N	
						Relations to other variables: 0	
						Consequences: 1	
Augmented reality telementoring (ART) platform (University of Nevada School of Medicine, Las Vegas, NV, USA)	Russo et al.^30^	Basic skills	Randomised control trial	Traditional mentoring	n = 19 (medical students)	Content: N	2
						Response processes: 0	
						Internal structure: 0	
						Relations to other variables: N	
						Consequences: N	
	Vera et al.^31^	Basic skills	Randomised control trial	Traditional mentoring	n = 18 (premedical and medical students)	Content: N	2
						Response processes: 1	
						Internal structure: 1	
						Relations to other variables: N	
						Consequences: N	
Hand-on Surgical Training (HoST) urethrovesical anastomosis (UVA) AR module (Roswell Park Cancer Institute and the State University of New York at Buffalo Virtual Reality Laboratory, New York, NY, USA)	Chowriappa et al.^32^	Urology	Randomised control trial	Traditional self-study	n = 52 (residents and fellows)	Content: 1	2
						Response processes: 1	
						Internal structure: N	
						Relations to other variables: 0	
						Consequences: N	
eoSim (eoSurgicalÔ, Edinburgh, Scotland, United Kingdom)	Hennessey^33^	Basic skills	Prospective observational	Box trainer	n = 24 (medical students and senior surgical trainees/consultants)	Content: 2	n/a
						Response processes: N	
						Internal structure: N	
						Relations to other variables: 2	
						Consequences: 0	
Brother AiRScouter WD-200B AR glasses (Brother International Corp, Bridgewater, NJ, USA)	Huang et al.^34^	Vascular	Prospective observational	Box trainer	n = 32 (physicians, sleep therapists and respiratory technicians)	Content: N	2
						Response processes: 1	
						Internal structure: 0	
						Relations to other variables: N	
						Consequences: 0	
NAV1 SinusTracker (KARL STORZ SE and Co. KG, Tuttlingen, Germany)	Linxweiler et al.^35^	Otolaryngology	Randomised control trial	Conventional navigation software	n = 100 (junior and senior surgical trainees)	Content: N	3
						Response processes: 1	
						Internal structure: 0	
						Relations to other variables: 1	
						Consequences: 1	
Modelled Anatomical Replica for Training Young Neurosurgeons (MARTYN) head (Royal College of Surgeons of England, London, UK) and custom And unsure custom software	Marcus et al.^36^	Neurosurgery	Randomised control trial	No image guidance or triplanar display of axial, sagittal and coronal images	n = 50 (novice trainees without endoscopic surgical experience)	Content: N	2
						Response processes: 1 internal structure: 2	
						Relations to other variables: 0	
						Consequences: 0	
PIÑATA, Optical See-Through AR (OST-AR) (Instituto Superior Técnico, Universidade de Lisboa, Portugal)	Mendes et al.^37^	Vascular	Prospective observational	Box trainer	n = 18 (novice medical residents and attending specialists)	Content: 2	2
						Response processes: 1	
						Internal structure: 1	
						Relations to other variables: 2	
						Consequences: 1	
A virtual interactive presence (VIP) platform (VIPAAR, Birmingham, Alabama)	Ponce et al.^38^	Orthopaedics	Prospective observational	Standard procedure performed in clinical setting	n = 15 (attending and resident surgeons)	Content: N	3
						Response processes: N	
						Internal structure: 0	
						Relations to other variables: 1	
						Consequences: 0	
VatSim-XR (AR mode) and Unity ARKit (Apple Inc, Cupertino, CA, USA)	Qin et al.^39^	Basic skills	Prospective observational	Box trainer, MR trainer, VR trainer	n = 32 (novice trainees (3-6 yrs experience and expert trainees (11-30 yrs experience)	Content: 2	2
						Response processes: 0	
						Internal structure: N	
						Relations to other variables: 2	
						Consequences: N	
Epson Moverio BT-200 Smart Glasses (Epson America, Inc, Long Beach, CA, USA)	Rochlen et al.^40^	Vascular	Prospective observational (survey)	Procedure without AR assistance	n = 40 (medical students, residents, fellows and nurse anaesthatists)	Content: N	1
						Response processes: N	
						Internal structure: 0	
						Relations to other variables: 2	
						Consequences: 0	
FluoroSim (Royal National Orthopaedic Hospital, Stanmore, London, UK)	Sugand et al.^41^	Orthopaedics	Randomised control trial	Traditional self study	n = 45 (medical students)	Content: 1	3
						Response processes: 1	
						Internal structure: 2	
						Relations to other variables: 2	
						Consequences: 1	
MicronTracker2 (Claron Technologies, Toronto, ON, Canada)	Sutherland et al.^42^	Neurosurgery	Survey	Standard procedure performed in clinical setting	n = 10 (medical students and residents)	Content: 2	1
						Response processes: N	
						Internal structure: N	
						Relations to other variables: 0	
						Consequences: 1	
Context-Aware Mobile Mix Reality Assistive Device headset (Juxtopia, Baltimore, Maryland)	Wilson et al.^43^	Trauma	Randomised control trial	Traditional self study	n = 34 (medical students)	Content: 1	2
						Response processes: 1	
						Internal structure: N	
						Relations to other variables: N	
						Consequences: 0	
Osterhaut Design Group R7 Smartglasses™ (San Francisco, California)	Yong et al.^44^	Otolaryngology	Prospective observational	Traditional self study	n = 7 (residents)	Content: N	1
						Response processes: N	
						Internal structure: N	
						Relations to other variables: 1	
						Consequences: 0	
Fundamentals of Laparoscopic Surgery (FLS) module (Society Of American Gastrointestinal and Endoscopic Surgeons, Los Angeles, CA, USA)	Zahiri et al.^45^	Basic skills	Randomised control trail	Box trainer	n = 20 (premedical students)	Content: 1	1
						Response processes: N	
						Internal structure: N	
						Relations to other variables: N	
						Consequences: 0	
Vuzix 920AR goggles (Vuzix Corp, Rochester, NY, USA)	Abhari et al.^46^	Neurosurgery	Prospective observational	Conventional 2D environment	n = 21 (novice medical students, surgical residents and fellows)	Content: 2	2
						Response processes: N	
						Internal structure: N	
						Relations to other variables: 1	
						Consequences: N	
MARVIS (Medical AR VISualizer) Warsaw University of Technology, Poland)	Gierwiało et al.^47^	Basic skills	Prospective observational	3D CT image	n = 25 (medical students and senior surgeons)	Content: 2	2
						Response processes: 0	
						Internal structure: N	
						Relations to other variables: N	
						Consequences: N	
Projector Method (Microprojector (MBP200; Samsung Electronics, Seoul, Korea) attached to probe from ultrasound device (LogiQ-e; GE Medical Systems, Shenzhen, China))	Jeon et al.^48^	Vascular	Randomised control trial	Standard procedure perfomed without AR device	n = 20 (medical trainees, sleep therapists and respiratory technicians)	Content: N	2
						Response processes: 1	
						Internal structure: 0	
						Relations to other variables: N	
						Consequences: 0	
Unnamed prototype made up of ascension 3D guidance trakSTARTM 6-DOF, artificial large left femur (sawbones part no. 1129), personal computer, drill with guide wire (Graduate College the University of Iowa Iowa city, Iowa)	Johns^49^	Orthopaedics	Prospective observational	Standard training without AR device	n = 8 (orthopaedic residents)	Content: 2	2
						Response processes: 0	
						Internal structure: 1	
						Relations to other variables: 2	
						Consequences: 2	
Unnamed smartphone AR application (Unity 2018.1, Unity Technologies, San Francisco, CA, USA) and Vuforia SDK, PTC, Boston, MA, USA)	Hecht et al.^50^	Cardiothoracic	Prospective observational	CT guided freehand	n = 11 (interventional radiologists, physicians and non-physicians)	Content: N	2
						Response processes: 0	
						Internal structure: N	
						Relations to other variables: N	
						Consequences: 0	
Unspecified prototype (department of gynecological surgery, clermont-ferrand, France)	Bourdel, N., et al.^51^	Gynaecology	Randomised controlled trial	MRI guidance	n = 60 (residents)	Content: 1	2
						Response processes: 1	
						Internal structure: 1	
						Relations to other variables: 1	
						Consequences: N	

## Results

**Figure 1.**
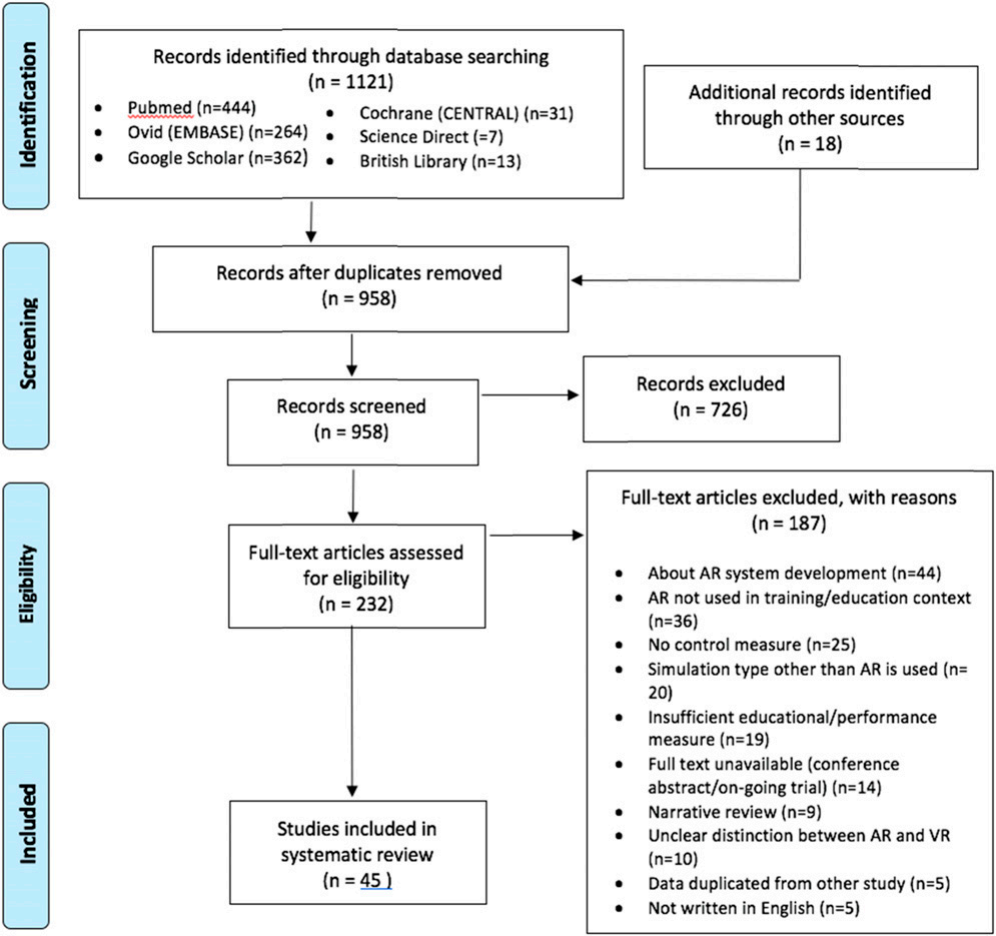
PRISMA flow diagram adapted from PRISMA statement.^3^

A total of 1132 studies were retrieved from the search, of which 173 were removed as duplicates and 726 were excluded following title and abstract review. A further 187 studies were excluded after full text examination, as summarised in Figure 1. The remaining 45 studies were included in the data analysis and categorised by simulator type, the details of the which are summarised in Table 1.

A detailed analysis of the results is given below.

### Microsoft Hololens

Seven studies covered the use of the Microsoft HoloLens (Microsoft Corp, Redmond, WA, USA). The HoloLens is an augmented reality head mounted display (ARHMD). The smart-glasses allow for a high level of user interactivity through the use of sensual and natural interface commands with gaze, gesture and voice (‘GGV’) inputs.

A prospective observational study on ureteroscopy revealed use of the HoloLens improved procedural times and OSAT scores for trainees, and 95% of participants agreed that the HoloLens had a role in surgical education.^7^ Another such study under neurosurgery discovered that trainees identified drill position and angle faster and more accurately with the HoloLens in the context of burr hole localisation, when compared to standard techniques.^8^ When used for cholecystectomy training, it was found that the HoloLens significantly improved economy of movement and error rates along with overall user performance.^9^ In the context of urogynaecological surgery, surgical residents’ knowledge, confidence and self-perceived preparedness scores increased significantly from the baseline.^11^ Similarly, for pre-op planning for lung adenocarcinoma, it was found that the HoloLens resulted in a shorter response time, more positive emotions and less cognitive load and effort.^12^ Another study reviewed the use of the HoloLens for surgical telementoring in trauma found no significant difference between the AR and the control group. While participants found the HoloLens made task completion easier, it resulted in a longer task completion time.^13^

A randomised control trial for orthopaedic training demonstrated that the use of AR led to a faster learning curve to achieve the same overall level of competency. While no participants favoured the use of the HoloLens as a training method by itself, 83% expressed that the combination of AR alongside their standard education would be their ideal learning strategy.^10^

Across these 7 studies, the HoloLens was afforded a collectively strong validity rating: content = 2; response processes = 1; internal structure = 1; relations to other variables = 2; consequences = 2. The overall LoE was 3.

### System for Telementoring with Augmented Reality

System for Telementoring with Augmented Reality (STAR) (Indiana University School of Medicine, Indianapolis, IN, USA) is an ARHMD that projects the operative instructions directly onto the wearer’s field of view. A total of 4 studies evaluated this system; 3 in the context of trauma and the other for basic surgical skills. The first study found that participants using STAR for leg fasciotomy simulations received a greater individual performance score, demonstrated fewer errors and increased confidence.^14^ Two studies reviewed the role of STAR for cricothyroidotomies. In both the randomised control trial and the prospective observational study, the utilisation of STAR received higher scores in all performance metrics.^15,16^For basic surgical skills teaching, STAR was found to cause fewer focus shifts and less placement error. There was no significant difference in task completion time for an abdominal incision task when compared to the conventional system, but port placement was 19% longer.

Overall, STAR was given a strong validity in most parameters: [content = 1; response processes = 2; internal structure = 1; relations to other variables = 2; consequences = 2; LoE = 2].

### Immersivetouch System

The ImmersiveTouch System (ImmersiveTouch, Inc, University of Illinois, Chicago, IL, USA) utilises specialised glasses and a robotic stylus to immerse the user in an interactive 3D environment with haptic feedback.^52^ Three prospective observational studies evaluated this simulation platform in the context of neurosurgery. The first study involved thoracic pedicle screw placement training and found a non-significant reduction of failure rate and indicated a trend toward learning retention, based on the improvement from practice to test sessions.^23^ The second study also indicated ImmersiveTouch ‘jump starts the learning curve’ when used for ventriculostomy training.^24^ The last study was also conducted for ventriculostomy training and disclosed similar findings; the AR group was more likely to succeed on their first attempt in the clinical environment.^22^

The overall performance of ImmersiveTouch in terms of validity and translational outcomes is promising. It reached strong validity in both content and internal structure and a high LoE: content = 2; response processes = N; internal structure = 2; relations to other variables = 1; consequences=1 and LoE = 3.

### Promis AR Simulator

The ProMIS AR simulator (Haptica, Dublin, Ireland) retains all the qualities of a traditional box trainer, but has the additional benefit of providing objective feedback on performance measures. Four prospective observational studies used ProMIS to assess and train basic laparoscopy skills. All studies showed an overall improvement and displayed higher scores in the ProMIS compared the control groups.^18,20^ However, in 1 study, this was thought to be due to repetition and familiarity with the tasks, and another found that global satisfaction was better for the cadaver model.^19,21^

Overall, ProMIS had a higher LoE and a strong validity of content but scored poorly in the other parameters: [content = 2; response processes = 1; internal structure = 1; relations to other variables = 1; consequences = 1; LoE = 3].

### Google Glass

The Google Glass, also simply known as Glass, is a pair of lightweight smart glasses that have been extensively trialled in medical research. In the case of this review, 2 studies were found that described their use in urology and basic surgical skills. The first was conducted as a prospective observation survey, where 81% of participants wanted the AR technology to be incorporated into their residency training programs and 93% felt that this had a place in the operating room.^26^ The second was a randomised control trial wherein Google Glass produced similar results to the control group. It was concluded that ARHMD were less useful but more enjoyable when utilised in learning.^27^

Although the LoE shows a positive level of translational outcomes, Google Glass rated poorly in all validity parameters: content = N; response processes = 1; internal structure = 1; relations to other variables = 1; consequences = 0 and LoE = 2.

### Perk Station

The Perk Station (National Alliance for Medical Image Computing (NAMIC), Boston, MA, USA), simulator for percutaneous surgery training, along with its successor, the Perk Tutor (Queen’s University, Kingston, ON, Canada), was described in 2 randomised control trials. The studies found that the AR simulator produced less tissue damage and a better success rate than the comparison in both orthopaedic and neurosurgery training contexts.^28,29^ The orthopaedic participants took a longer time to adjust to the free hand technique than their counterparts from the control group, but their accuracy and success rate remained high.

Overall, Perk Station scored a positive LoE but was rated relatively low on all validity parameters: content = 1; response processes = 1; internal structure = 0; relations to other variables = 0; consequences = 1 and LoE = 2.

### Art Platform

ART Platform (University of Nevada School of Medicine, NV, USA) is a simulator designed to overlay the instruments of a mentor onto the trainee’s laparoscopic monitor. Two randomised control trials were performed which demonstrated faster skill acquisition, quicker task completion time, fewer errors and a shorter, steeper learning curve compared to the traditional methods.^30,31^

However, despite the positive translational outcomes, ART Platform also scored poorly in terms of validity: content = N; response processes = 1; internal structure = 1; relations to other variables = N; consequences = N and LoE = 2.

### Other AR Simulators

VatSim-XR (AR mode) and Unity ARKit (Apple Inc, Cupertino, CA, USA) is a simulator model capable of adopting several different simulation modes – AR, VR and Mixed Reality (MR). Each mode was tested against a traditional box trainer and found that AR provided the most balanced training environment with regards to fidelity and accuracy. However, the box trainer and MR trainer demonstrated a superior realism, 3D perception and immersive surgical performance. The VatSim-XR scored strongly (rating of 2) in both content and relations to other variables, but further development is required to provide better outcomes than the traditional method of training.^39^

The eoSim (eoSurgicalÔ, Edinburgh, Scotland, United Kingdom) is a laparoscopic surgery simulator and was used in a prospective observational study to train basic surgical skills. It showed a strong validity in content and relation to other variables and showed correlation with the control group. The eoSim was deemed a useful educational tool through Likert Scale analysis and awarded an LoE of 2, but participants felt that the control method was more beneficial, overall.^33^

PIÑATA, Optical See-Through AR (OST-AR) (Instituto Superior Técnico, Universidade de Lisboa, Portugal) relies on medical dummies which simulate the surface anatomy of veins and arteries and an OST-AR interface for needle insertion and central venous access. The PIÑATA had strong validity in control and relations to other variables (rating of 2) and scored 1 in all other parameters. Participants in the study strongly favoured the implementation of PIÑATA into traditional surgical training and an LoE of 2 was awarded.^37^

Two studies under orthopaedics described simulators with a relatively strong validity. The FluoroSim consists of a virtual environment FluoroSim (Royal National Orthopaedic Hospital, Stanmore, London, UK), a haptic device, 3D printed drill and a VR headset.^41^ It was used in a randomised control trial to assess dynamic hip screw guidewire insertion. Participants using the FluoroSim outperformed the control group in regard to procedural time, number of radiographs and guidewire retries. While there was an improved baseline of the learning curve and after a 1-week washout period, the short duration of the study could not provide evidence for a longer-term skill retention. Additionally, this improvement was not significantly greater than that observed in the control group.

Similarly, a novel prototype simulation was described in,^49^ where there was no significance difference between the AR and control group when assessing wire navigation. While both simulators showed strong validity in content and relations to other variables, as they showed no significant difference in overall performance measures compared to the control groups, they cannot be recommended over traditional training without further studies.

Several other augmented reality simulators have also been depicted in the literature, including 4 under vascular surgery describing central venous catheterisation^37,40,48^; 1 under urology, training ureterovesical anastomosis^32^; 2 for otolaryngology for functional endoscopic sinus surgery and dissection^21,34,44^; 3 under neurosurgery for tumour resection planning, spinal needle insertion and identification of a basilar tip aneurysm^46^; 1 under orthopaedics for surgical telementoring of arthroscopic shoulder procedures^38^; under basic laparoscopy skills, including suturing, peg transfer and needle insertion^33,39,45,47^; under trauma for tension pneumothorax^43^; 1 under gynaecology and 1 under cardiothoracic for needle insertion.^50^ However, all of these were stand-alone reports which only suggested strong validity in a maximum of 1 validity parameter and did not have a LoE above 2. Therefore, they could not provide strong evidence that AR produces better outcomes when implemented within surgical training.

The only exceptions were the ‘A virtual interactive presence (VIP) platform’ (VIPAAR, Birmingham, Alabama) and the NAV1 SinusTracker (KARL STORZ SE and Co KG, Tuttlingen, Germany), which both achieved an LoE of 3. However, neither simulator outperformed the control group and so could not be recommended over traditional methods

## Discussion

This systematic review focused on clinical trials which evaluated the impact of AR on performance and educational measures of trainees during surgical training. It has described 45 studies which cover a wide range of surgical specialties and AR training models.

The Microsoft HoloLens, STAR, and ImmersiveTouch systems definitively had an overall positive effect on performance measures for surgical trainees when compared to traditional means, especially when used as an addendum to the standard methods of surgical education. Scoring highly in content validity and LoE, ProMIS could be deemed a useful simulator tool. However, lacklustre performance across the other parameters necessitates further development and research before determining the strength of its outcomes. Likewise, while the integration of the Google Glass has received positive feedback, there was insufficient evidence to suggest that it improves the performance measures of surgical trainees, due to its poor rating across validity parameters. This was also the case regarding the Perk Station and ART platforms, which both scored highly in LoE but achieved less promising validity ratings.

Of all the AR models, only the HoloLens, STAR, ImmersiveTouch, ProMIS, Google Glass, Perk Station and ART Platform were described by more than 1 study. Of those, the HoloLens scored the overall highest validity across all parameters. This was followed by ImmersiveTouch and STAR, which both showed relatively strong validity across all parameters; a high LoE of 3 and 2, respectively.^16,17,22^ While the ProMIS and Google Glass were also assessed by more than 1 study, there was variation in the strength of the study methodology and level of bias. Therefore, their value as a training tool remains inconclusive until further investigation. The VatSim-XR (AR mode) and Unity ARKit, eoSim, PIÑATA, FluoroSim and Unnamed prototype were also rated relatively highly across the validity parameters and LoE. However, they were all in their early stages of development and were not ready for commercial use in surgical training.

It would be premature to crown the Microsoft HoloLens as the superior AR tool without taking into account reporting bias. As the HoloLens was described by more studies, more areas of validity had the opportunity to be assessed. Thus, despite the Microsoft HoloLens having the highest amount of supporting evidence, this is not necessarily a reflection on its performance as an educational tool when compared to the other models. Rather, this emphasises the importance of validity assessment in study design.

Messick’s criteria presents a more comprehensive study of validity when compared to past methodologies by subsuming and presenting a unified approach of previous theories. The majority of studies lacked a standardised validation process and were purely descriptive in their analysis, with 91% having no discussion of at least 1 validity criterion. ‘Content’ was the best assessed criterion, with 22% of studies achieving a score >1; ‘Response processes’ was the least reviewed criterion, only achieving a score >1 in a singular study. ‘Relations to other variables’ was the most discussed parameter. Although this measure is useful to illustrate the construct validity and realism of a model, on its own, this criterion does little to evaluate the simulations’ educational impact. In general, it is to be expected that novices perform poorly when compared to experts; however, the effectiveness of AR simulation in bridging this gap remains ambiguous without considering the other variables.

The lack of discussion on certain parameters may be attributed to the reliance of many studies on outmoded definitions of validity when approaching their research design. For instance, several studies quoted achieving ‘face validity’ as a goal.^53-56^ However, face validity is recognised as being a poor measure due to the subjective nature of ‘perceived realism’ and lack of quantifiability, thus diminishing the overall impact of results.^57^

On review, although the studies evaluated a number of criteria, there were several confounding factors. For example, many studies did not consider the learning curve participants may have experienced when familiarising themselves with the new AR technology. Improvement in performance could well have been attributed to increased confidence when navigating the simulation technology, as opposed to the direct result of any educational impact provided by the AR model itself. Secondly, possibility of any additional training the participants may have acquired outside of the simulation environment was not accounted for within the study designs. A common theme amongst the included papers was the use of surveys to assess the confidence level of participants and whether they believed AR had a place in the clinical environment. While it has been well established that trainees’ confidence is not an insignificant contributor to improved surgical competence, a large proportion of participants were either medical students or novice level surgical trainees.^58^ Input from more expert level surgeons may have provided a better insight into the pragmatism of adopting the AR model into the clinical training environment, consequently negating the risk of inexperience skewing the results.

Appraisal of translational outcomes was an area which lacked extensive testing. The highest LoE recorded was 3, which only 7 AR platforms achieved.^8,21,22,25,35,38,41^ Collateral effects, especially the financial benefits of the AR technology, were not described in detail. However, platforms such as the Projector Method alluded to the latter as it produced improved performance measures in surgical trainees, despite its relatively primitive and cheap design.^48^ It can also be argued that the cost-saving effect is inherent, if intervention with the AR modality results in enhanced surgical competence in trainees. Increased surgical competence reduces the risk of complications, therefore saving expenses on consequential prolonged hospital stays and repeat surgeries.

There are several limitations to this review. Firstly, within the studies included and secondly, with the nature of the review itself. The included studies had a large degree of heterogeneity in study design and outcome measures. Out of the 45, only 14 studies were randomised control trials, and only 9 studies overall included a sample size of over 50. Therefore, the quality of research used in this review was generally poor. Moreover, many of the studies focused on the development of the AR model and while the construct validity tended to rate relatively high, many of the other parameters rarely rated above 1. Only 7 studies assessed the simulation in a clinical environment. Of those, the duration of the research was too short to measure long term translational outcomes, such as skill retention. Furthermore, their limited scope did not allow for assessment of behavioural changes in the clinical environment in regard to patient safety and cost saving.

As with any qualitative systematic review, the study design is inherently biased as there is no objective process for measuring validity and translational outcomes of studies. Further, there is the unavoidable flaw of reporting bias due to the search criteria used (ie, only English language studies were included, choice of database and keywords). We also acknowledge that the terms ‘mixed reality’ and ‘augmented reality’ are sometimes used interchangeably. Had we included the term ‘mixed reality’ in our search string, it is possible that this study may have yielded a different number of papers.

It is pertinent to note that the platforms included in this study were at different stages of developmental design. For example, the HoloLens and ImmersiveTouch systems are well-established systems with widespread commercial use, whereas other platforms used in this study were only prototypes.^48-51^ Disparities in the technological advancement of the modalities may have skewed the results in favour of the more accessible platforms. However, even the preliminary results for the prototype platforms were in favour of implementing AR in surgical training.

Technical skill acquisition is the core around which surgical training has been centred. With the influx of innovative new technology seeping into the modern operating theatre, surgeons are faced with more stimuli and digital ouput than the tradition methods of surgical training prepare them for. Augmented reality creates the opportunity for that gap to be bridged - by the merging of the digital and physical world, input can be selected and managed, thus reducing the cognitive load and improving situational awareness while performing surgery, a skill which has been neglected in medical school teaching.^12,59^

Allowing surgeons to become familiar with immersive technology facilitates for skill acquisition in a low-risk environment, making room to learn from errors and establish muscle memory. Establishing AR in surgical training has the possibility for widespread and long-term benefits: skill acquisition and retention, reduced cost and better patient outcomes with more competent surgeons.

## Conclusion

In conclusion, AR technology has fundamentally changed the way in which trainees can acquire mastery of surgical skills. Use of AR has opened up the realms of exciting opportunities for innovation in medical education. After appraisal of the existing systems, it was revealed that the Microsoft HoloLens has shown the most promising results - both in terms of validity and level of effectiveness – when used in surgical training to produce improved performance measures. In terms of the other simulator models, there needs to be further research, with stronger study designs in order to justify the use of AR in surgical training. Overall, this review has highlighted the importance for future studies in this area to incorporate a more rigorous validation process in their methodologies. Likewise, potential areas of investigation could be the evaluation of long-term skill retention, financial impacts and trainee behavioural changes.

Despite issues with the quality of studies, the early results are largely in favour of the integration of AR simulation in surgical training. Simulation technology has brought forth a plethora of possibilities in the delivery of medical education, and continued advancement and innovation indicate a bright future for AR in this sphere.

## Supplemental Material

Supplemental Material - The Role of Augmented Reality in Surgical Training: A Systematic Review Click here for additional data file. Supplemental Material for The Role of Augmented Reality in Surgical Training: A Systematic Review by Dhivya Suresh, BSc (Hons), Abdullatif Aydin, BSc (Hons), MBBS, PhD, Stuart James, BSc (Hons), MBBS, Kamran Ahmed, PhD, and Prokar Dasgupta, MSc, MD, FEBU in Surgical Innovation

## Appendices

### Appendix 1

**Appendices Appendix 1Table A1.** Framework to Evaluate Validity Adapted From Ref. [4,5]

Parameter	Criteria	Example	Rating	
Content	Test items are relevant and representative of the intended construct	Using expert opinions to ensure all domains are accurately represented	N =	No discussion of instrument content (includes simply listing items without justification)
			0 =	Discussion but no data
			1 =	Listing assessment themes with little or no reference to a theoretical basis, or a poorly defined process for creating and reviewing items
			2 =	Well‐defined process for developing instrument content, including both an explicit theoretical/conceptual basis for instrument items and systematic item review by experts alternatively, reference to a prior study on an assessment instrument that meets these criteria
Response process	Thought processes and actions of subjects and observers are made in accordance with the intended construct	Quality control of assessments, such as in standardizing test administration, minimizing examiner bias, and providing a specific test for the task	N =	No discussion. Merely disclosing response rates or numbers of respondents does not constitute evidence
			0 =	Discussion but no data. Discussing the impact of response rate on assessment scores, or speculating on the thought processes of learners, does not constitute evidence
			1 =	Minimal data regarding thought processes and analysis of responses. Description (without data) of systems that reduce response error, such as computer‐scored forms
			2 =	Multiple sources of supportive data, including critical examination of thought processes, analysis of responses for evidence of halo error or rater leniency, or data demonstrating low response error
Internal structure	Test scores across tasks can be reliably reproduced	Calculating interitem reliability and test-retest reliability	N =	No discussion
			0 =	Discussion but no data
			1 =	Factor analysis incompletely confirming anticipated data structure, or acceptable reliability with a single measure
			2 =	Factor analysis confirming anticipated data structure, or multiple measures of reliability. Variation in responses to specific items among subgroups (differential item functioning) can support or challenge internal structure depending on predictions
Relations to other variables	Test scores correlate with external, independent measures that share a theoretic relationship	Comparing scores between groups with different levels of experience in the tested skill	N =	No discussion
			0 =	Discussion but no data
			1 =	Correlation of assessment scores to outcomes with minimal theoretical importance, or unanticipated score correlations
			2 =	Correlation (convergence) or no correlation (divergence) between assessment scores and theoretically predicted outcomes or measures of the same construct. Such evidence will usually be integral to the study design, and anticipated a priori
Consequences	The impact of using the assessment	Determining the pass-fail score and considerations for the subject on obtaining a pass or fail, promotion, or privilege	N =	No discussion. Speculation on potential applications of the assessment does not constitute evidence
			0 =	Discussion but no data. Simply discussing the consequences of assessment (eg, data regarding usefulness or faculty approval) without linking this to validity does not constitute evidence
			1 =	Description of consequences of assessment that could conceivably impact the validity of score interpretations (although these impacts are not explicitly identified by the authors)
			2 =	Description of consequences of assessment that clearly impact on the validity of score interpretations, as supported by data and convincingly argued by the authors. Such evidence will usually be integral to the study design, and anticipated a prior

### Appendix 2

**Appendix 2Table B1.** Framework to Evaluate Translational Outcomes.^6^

Parameter	Definition	Example	Level of effectiveness
Internal acceptability	The trainee’s satisfaction with using the simulator	Favourable responses from feedback forms or post-training survey questionnaires	1
Contained effects	Changes in performance in the simulation context	Development of knowledge and/or skills as measured by the simulator tool	2
Downstream effects	Behavioural changes in the clinical context	Adopting safer patient-care practices	3
Target effects	Direct changes to patient outcomes	Reduced rates of surgical complications	4
Collateral effects	Changes on a wider, systemic level	Cost saving; skill retention	5
